# An Alarmin Role for P2Y_13_ Receptor during Viral-driven Asthma Exacerbations

**DOI:** 10.1164/rccm.202111-2571ED

**Published:** 2021-12-22

**Authors:** Adam J. Byrne, Sejal Saglani, Robert J. Snelgrove

**Affiliations:** National Heart and Lung Institute Imperial College London London, United Kingdom

An array of distinct, nonspecific stimuli may evoke a prototypic type 2 immune response in the airways. This is achieved through their shared capacity to induce the release of stress signals or “alarmins” from airway sentinels such as airway epithelial cells (AECs), which, in turn, function to initiate and amplify type 2 inflammation by acting primarily upon type 2 innate lymphoid cells and CD4^+^ (cluster of differentiation 4–positive) T-helper type 2 (Th2) cells (1). IL-33 and HMGB1 (high-mobility group box 1 protein) are two alarmins released from AECs fundamental to establishing a type 2 inflammatory response. Levels of IL-33 and HMGB1 are elevated in patients with asthma, correlating with Th2 inflammation and disease severity, whereas targeting these mediators in animal models ameliorates inflammation and pathology (1–5). Intriguingly, targeting HMGB1 diminishes IL-33–induced type 2 inflammation, suggesting that these mediators coordinate in a feed-forward circuit to amplify Th2 responses (6).

Although the importance of IL-33 and HMGB1 in driving type 2 inflammation is irrefutable, the mechanisms by which diverse stimuli converge to drive their release from AECs is less well defined. Both IL-33 and HMGB1 are constitutively expressed in the nuclei of AECs, and it is proposed that their extracellular availability results from passive release from necrotic or damaged cells or active cell death–independent secretion. Allergen-induced activation of TLR4 or protease-activated receptors and viral-induced necroptosis are all purported mechanisms driving the secretion of these alarmins (7–10). Increasing evidence suggests a signaling cascade whereby AEC exposure to these environmental stimuli elicits extracellular ATP accumulation and autocrine purinergic P2 receptor activation as an intermediary step in driving alarmin release (8, 11, 12); however, the precise receptor subtype responsible remained ambiguous. Genome-wide association studies have highlighted P2Y_13_-R, a purinergic receptor that is highly sensitive to ADP, as a risk factor for asthma (13). P2Y_13_-R is upregulated on AECs of allergen-exposed mice, and ADP administration was sufficient to induce IL-33 release and airway eosinophilia (13).

In this issue of the *Journal*, Werder and colleagues (pp. 300–312) provide compelling evidence implicating P2Y_13_-R as a key regulator of IL-33 and HMGB1 release (14). An analysis of human lung biopsies showed that the majority of AECs coexpress P2Y_13_-R, IL-33, and HMGB1. Exposure of AECs to HDM allergen induced the release of the P2Y_13_-R agonists, ADP and ATP, whereas subsequent pharmacological inhibition of P2Y_13_-R abrogated cytoplasmic translocation and release of IL-33 and HMGB1 to multiple aeroallergens or rhinovirus. Importantly, the authors showed that these effects were specific to P2Y_13_-R, as other purinergic receptor antagonists had no effect, whereas AECs from P2Y_13_-R^−/−^ mice showed reduced allergen-induced alarmin release. Consistent with *in vitro* findings, IL-33 and HMGB1 release into the airways of acute allergen–exposed mice was preceded by a rapid increase in ADP and/or ATP. Genetic or pharmacological inhibition of P2Y_13_-R inhibited this allergen-induced alarmin release and downstream inflammation. In an HDM-induced experimental asthma model, P2Y_13_-R antagonist administration again ameliorated Th2 inflammation and aspects of airway remodeling, but the effect on airway hyperresponsiveness was not assessed, and the benefit was only apparent when antagonist administration preceded allergen exposure, questioning the potential effectiveness of P2Y_13_-R antagonists in patients with established asthma. However, the authors subsequently demonstrated that therapeutic P2Y_13_-R antagonism ameliorated airway inflammation and promoted viral clearance in a mouse rhinovirus-induced asthma exacerbation model.

Although the authors demonstrate the importance of the P2Y_13_-R signaling cascade in dictating the release of IL-33 and HMGB1 from AECs, it would be prudent to determine whether this pathway is conserved in other cell types that produce these alarmins. Targeting P2Y_13_-R in an *in vivo* setting clearly impacted extracellular IL-33 and HMGB1, highlighting the physiological relevance of this pathway in specific models. However, P2Y_13_-R is upregulated on multiple cell types within the mouse lung after allergen challenge (13). Thus, it would be of interest to demonstrate that AECs are indeed the primary target of P2Y_13_-R–mediated IL-33 and HMGB1 release in these instances and/or whether this signaling network also defines release of alarmins from disparate cell types. Accordingly, the future generation and application of epithelial-specific P2Y_13_-R floxed mice would be informative. Furthermore, P2 purinergic signaling has been demonstrated to modulate the function of multiple cells, independent of IL-33 and HMGB1 release, and thus it would be helpful to know whether the amelioration of inflammation and pathology observed after inhibition of P2Y_13_-R in asthma and asthma exacerbation models is entirely attributable to diminished alarmin secretion.

**Figure 1.**
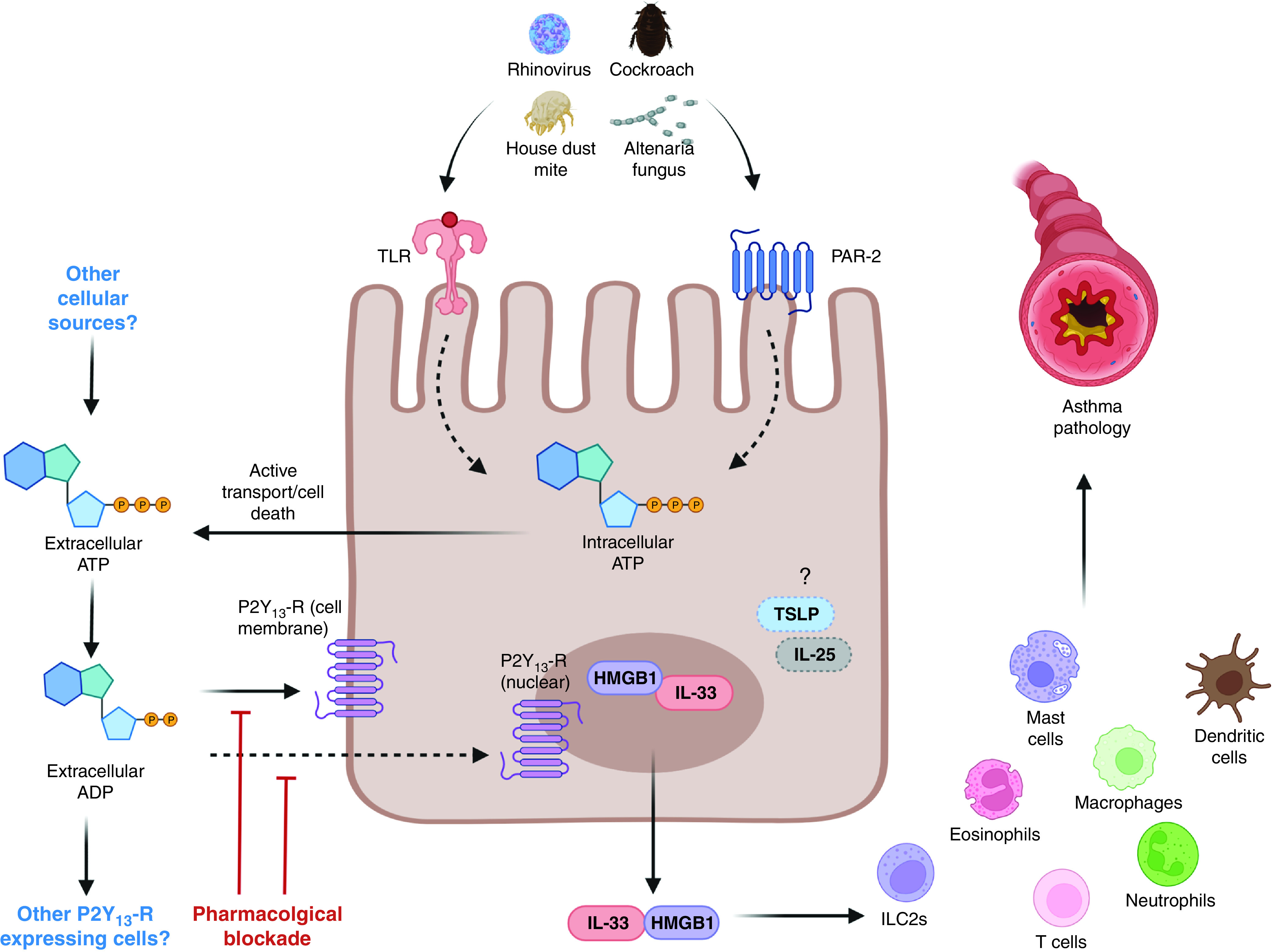
Can targeting purinergic receptors ameliorate asthma and associated exacerbations via regulation of airway epithelial cell alarmin release? Werder and colleagues demonstrate that the exposure of airway epithelial cells to allergens induces the release of the P2Y_13_-R agonists, ADP and ATP. P2Y_13_-R activation (transmembrane or nuclear) induces release of the alarmins HMGB1 and IL-33, which, in turn, can drive type 2 immunity and airway mucus secretion. Genetic or pharmacological manipulation of P2Y_13_-R inhibited alarmin release and downstream inflammation in models of allergen exposure and rhinovrus-driven exacerbation of allergic airway disease. Depicted are opportunities for therapeutic intervention. Questions remain regarding the source of ADP, which triggers AEC P2Y_13_-R, the cells that respond to these signals (AECs or other P2Y_13_-R–expressing cells), in addition to the role for other AEC-derived alarmins (e.g., TSLP or IL-25). AEC = airway epithelial cell; HMGB1 = high-mobility group box 1 protein; ILC2 = type 2 innate lymphoid cell; P2Y_13_-R = purinergic receptor Y_13_; PAR = protease-activated receptor; TLR = Toll-like receptor; TSLP = thymic stromal lymphopoietin.

An intriguing aspect of this study is that multiple stimuli all converge on P2Y_13_-R to mediate IL-33 and HMGB1 release from AECs. It seems probable that allergen or virus trigger ADP and ATP release from AECs and that ensuing autocrine signaling via P2Y_13_-R is the shared intermediary step in defining the release of these alarmins (Figure 1). However, respiratory syncytial virus can drive HMGB1 release from AECs via necroptosis (10); thus, it will be of interest to elucidate how disparate pathways align and contribute to the release of these alarmins in response to diverse stimuli in distinct physiological settings. Although the authors have convincingly demonstrated the importance of P2Y_13_-R signaling in governing the release of both IL-33 and HMGB1, it is unclear whether this mechanism is conserved for governance of release of other pro-Th2–inducing alarmins, such as IL-25 and TSLP. There is a level of redundancy in function of these alarmins, and thus determination of the universality of an intermediary ADP/ATP–P2Y_13_-R signaling step would be important when deciding therapeutic application in distinct settings.

Although the authors clearly demonstrate the central role for P2Y_13_-R in mediating IL-33/HMGB1 release from AECs, it should be noted that no observable difference was apparent between cells derived from healthy subjects or patients with asthma. Epithelial P2Y_13_-R expression was comparable between healthy donors and patients with asthma, whereas there was equivocal release of ADP/ATP after allergen provocation and of alarmin secretion after stimulation with ADP/ATP. There is clearly, therefore, more scope for exploration of the P2Y_13_-R pathway in larger cohorts of heterogeneous patients with asthma to infer whether differences in expression or function of this axis are a precipitating factor driving an aberrant Th2 response. There is a suggestion that nuclear expression of P2Y_13_-R is greater in asthmatic-derived AECs, which is potentially intriguing, but the significance of this observation is currently unclear. Nonetheless, P2Y_13_-R is seemingly an eminently druggable target in the context of type 2 inflammation and could hold added benefit over current strategies that seek to target IL-33, such as it operating as a common upstream regulator of multiple, partially redundant, pro-Th2 alarmins. Overall, the most promising clinical application for blocking P2Y_13_-R is likely for the treatment for rhinovirus-induced asthma exacerbations, in which pathological type 2 inflammation has been shown to be IL-33 dependent (15). In this context, the added advantage comes from not only reducing inflammation but also promoting viral clearance by eliminating the IL-33/HMGB1–mediated suppression of host IFNs.
